# Technical note: A comparison of in‐house 3D‐printed and commercially available patient‐specific skin collimators for use with electron beam therapy

**DOI:** 10.1002/acm2.14366

**Published:** 2024-04-26

**Authors:** Steven M. Herchko, Michael S. Rutenberg, Chris J. Beltran, Sridhar Yaddanapudi

**Affiliations:** ^1^ Department of Radiation Oncology Mayo Clinic Jacksonville Florida USA

**Keywords:** 3D printing, electron beam therapy, external beam therapy, patient‐specific devices

## Abstract

**Purpose:**

Skin collimation is a useful tool in electron beam therapy (EBT) to decrease the penumbra at the field edge and minimize dose to nearby superficial organs at risk (OARs), but manually fabricating these collimation devices in the clinic to conform to the patient's anatomy can be a difficult and time intensive process. This work compares two types of patient‐specific skin collimation (in‐house 3D printed and vendor‐provided machined brass) using clinically relevant metrics.

**Methods:**

Attenuation measurements were performed to determine the thickness of each material needed to adequately shield both 6 and 9 MeV electron beams. Relative and absolute dose planes at various depths were measured using radiochromic film to compare the surface dose, flatness, and penumbra of the different skin collimation materials.

**Results:**

Clinically acceptable thicknesses of each material were determined for both 6 and 9 MeV electron beams. Field width, flatness, and penumbra results between the two systems were very similar and significantly improved compared to measurements performed with no surface collimation.

**Conclusion:**

Both skin collimation methods investigated in this work generate sharp penumbras at the field edge and can minimize dose to superficial OARs compared to treatment fields with no surface collimation. The benefits of skin collimation are greatest for lower energy electron beams, and the benefits decrease as the measurement depth increases. Using bolus with skin collimation is recommended to avoid surface dose enhancement seen with collimators placed on the skin surface. Ultimately, the appropriate choice of material will depend on the desire to create these devices in‐house or outsource the fabrication to a vendor.

## 1 INTRODUCTION

**FIGURE 1:**
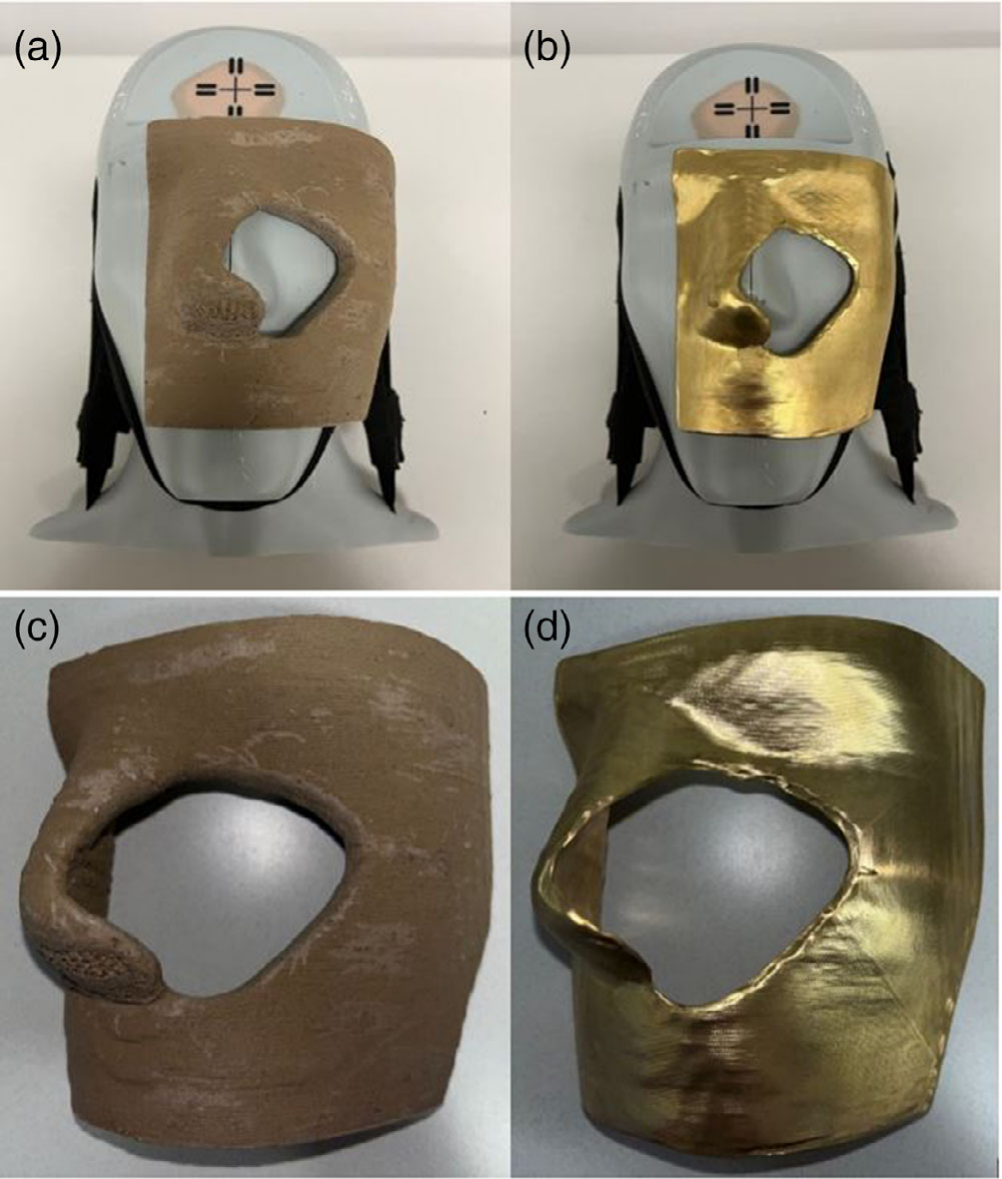
(a) 3DPB and (b) MB collimation devices on a phantom. Close‐up view of (c) 3DPB and (d) MB collimation devices.

Electron radiotherapy poses unique challenges and opportunities due to the nature of electron beams and their interaction with matter. Skin shielding is a critical aspect of electron radiotherapy, as it reduces the dose to the underlying healthy tissue while maintaining the targeted dose to the tumor. Achieving this balance is essential to maximize treatment efficacy. Traditionally, skin shielding has relied on conventional methods such as lead or Cerrobend blocks.^1^, ^2^, ^3^, ^4^ The toxic composition of lead and Cerrobend has led to the implementation of stringent regulatory requirements and costly infrastructure. These traditional skin shielding methods present limitations in terms of customization, geometric complexity, and ease of fabrication. The advent of additive manufacturing, 3D printing, has revolutionized the field of radiation oncology by offering innovative solutions for patient‐specific treatment accessories.^5^, ^6^, ^7^ The ability to create intricate, customized designs with high precision has opened doors to a new era of skin shielding possibilities. Two types of materials, high‐density composite 3D printing filaments, and machined brass, have emerged as promising candidates for skin shielding applications in electron radiotherapy (Figure 1).

Bronzefill (colorFabb, Belfeld, The Netherlands) is a composite filament formed by blending polylactic acid (PLA) with a substantial proportion of finely powdered bronze. The inherent high‐density of bronze allows for the attenuation of low energy electron beams with reasonable device thicknesses. Craft et al. published the first case of a patient treatment using a custom 3D‐printed skin shield using Bronzefill, and their methods have been implemented at our institution for patient‐specific skin shielding.^8^

Machined brass, a copper‐zinc alloy renowned for its exceptional durability, thermal conductivity, and machinability, offers another approach to skin shielding. Machined brass enables the creation of skin shields with reduced thickness while maintaining structural integrity. The controlled manufacturing process of brass lends itself to systematic quality assurance measures, enhancing its appeal in clinical applications. Brass has been used for other applications in radiation oncology, such as spatially fractionated radiation therapy, bolus, and compensators.^9^, ^10^, ^11^

This study provides a comprehensive comparative analysis of 3D‐printed Bronzefill (3DPB) and machined brass (MB) skin shields for electron radiotherapy treatments. We present the dosimetric characteristics of the skin shields. Dosimetric characteristics encompassing surface dose and dose distribution beneath the shield are examined. Clinical applicability considerations include design, ease of handling, patient comfort, and quality assurance protocols. These clinical considerations, while important, are beyond the scope of this technical report. All brass materials used in this work were ordered from .decimal (.decimal Inc., Sanford, Florida, USA), and all 3D printing was done on an AXIOM DUAL Extruder 3D Printer (Airwolf 3D, Las Vegas, Nevada, USA). The settings recommended by Craft et al. were used as a starting point for the 3D printer and modified to create adequate print quality while maintaining achievable print times.^8^

## 2 METHODS

All measurements were performed on a Varian TrueBeam linear accelerator (Varian Medical Systems, Palo Alto, California, USA) with solid water. Attenuation measurements were performed using a parallel plate ionization chamber placed at both the surface of the solid water and at the depth of maximum dose (1.3 cm for 6 MeV and 2.1 cm for 9 MeV) as described in AAPM Task Group 25.^12^ Calibrated GafChromic EBT3 film (Ashland, Wayne, New Jersey, USA) was used for the surface dose and dose plane analysis. Film analysis was performed using the FilmQA Pro software (Ashland Inc., Wayne, New Jersey, USA).

### 2.1 Attenuation measurements

To determine the amount of material needed to appropriately attenuate 6 and 9 MeV electron beams, varying material thicknesses were placed in the beam path to measure the transmission through the attenuating material. The surface of the solid water was placed at 100 cm source‐to‐surface distance (SSD), and attenuating material was placed on the surface of the solid water. 3DPB thicknesses of 0.4, 0.6, 0.8, 1.2, and 1.6 cm and MB thicknesses of 0.2, 0.4, 0.6, 0.8, and 1.0 cm were used for these measurements.

### 2.2 Relative dose plane analysis

Once the desired collimation thickness was determined based on attenuation measurements, flat surface collimators of both 3DPB and MB were created with a 4.0 cm diameter circular aperture. Dose planes for 6 MeV were measured at depths of 0.0, 0.2, 0.5, 1.0, 1.3, and 2.3 cm, and dose planes for 9 MeV were measured at 0.0, 0.3, 0.6, 1.1, 2.1, and 3.1 cm.

**FIGURE 2:**
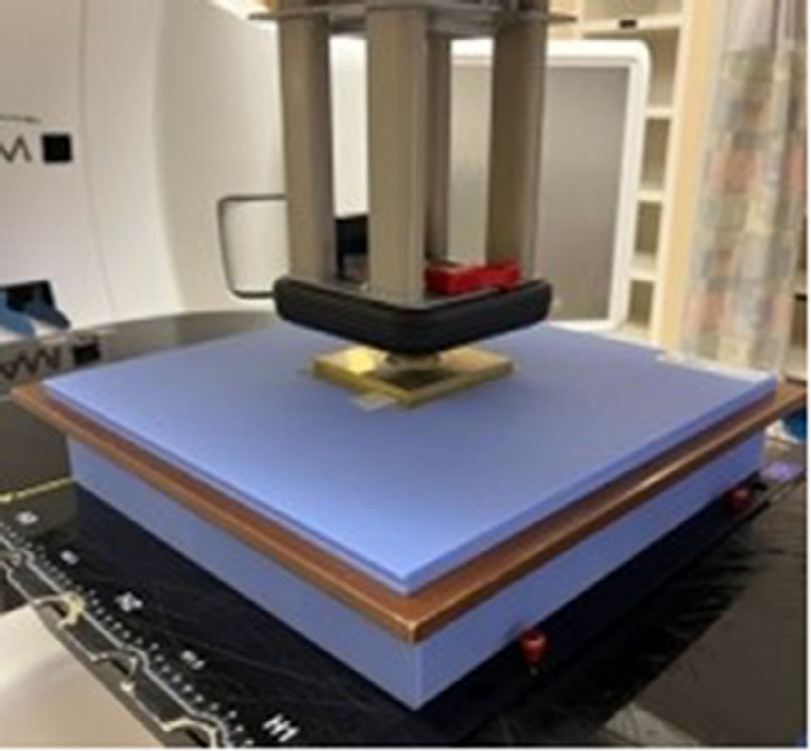
Experimental setup for relative dose plane and surface dose measurements with MB collimation. A flat collimator of the appropriate thickness was placed on the surface of the solid water with Gafchromic film placed at various depths. Measurements were repeated with appropriate collimators for both 6 and 9 MeV using both 3DPB and MB.

A 6 × 6 cm^2^ electron applicator and a 5.0 cm diameter circular electron block were used for the measurement. The light field projected a uniform 0.5 cm margin beyond the aperture of the surface collimator (Figure 2). Additional measurements with no surface collimator and a 4.0 cm diameter circular electron block were performed for comparison with the surface collimation results.

The field width, flatness, and penumbra were measured for all dose planes and calculated in FilmQA Pro using relative dose profiles and the calculation protocols defined in AAPM Task Group 45.^13^ Films were scanned at a resolution of 96 dpi, and dose profiles were generated with a path width of 5 pixels.

### 2.3 Surface dose measurements

Surface dose measurements were performed by measuring absolute dose planes at the phantom surface using the setup described previously. All films received 500 monitor units (MUs), and measurements were performed with a 5.0 cm diameter circular electron block with and without bolus material. For bolus measurements, a 4.0 cm diameter circle with a thickness of 0.5 cm Superflab (RPD Inc, Albertville, Minnesota, USA) was cut and placed in the 4.0 cm diameter circular aperture created by the surface collimator. The absolute dose was determined by taking the mean value of a 1.0 cm^2^ circular region of interest (ROI) centered in the radiation field, and the relative surface dose was calculated by dividing the dose with skin collimation by the dose without skin collimation.

### 2.4 Clinical workflow

To obtain patient‐specific 3DPB and MB devices, a CT simulation is performed with the desired treatment area defined on the patient's skin surface with a radiopaque wire. A device with the appropriate thickness is created in the treatment planning system (TPS) as a structure to treat the desired area while shielding adjacent areas. For 3DPB devices, the structure is exported in DICOM format to MIM (MIM Software Inc., Cleveland, Ohio, USA) where it is converted to a .stl file. The .stl file is then imported and converted to the appropriate 3D printing file type in the Simplify3D printing software (Simplify3D, Cincinnati, Ohio, USA). For MB devices, the desired structure is exported in DICOM format from the TPS to p.d (.decimal Inc., Sanford, Florida, USA) where it is ordered and sent to the vendor for fabrication. Quality assurance for the devices is performed by visually inspecting the device, measuring the device thickness with calipers, and measuring the weight of the device for comparison with the expected weight based on the expected volume and density.

### 2.5 Patient treatment in vivo dosimetry

The first patient at our institution treated with a MB device is described. The patient was treated with 6 MeV electrons for a right temple lesion adjacent to the patient's right eye. A 0.5 cm MB device was designed to conform to the patient's anatomy with an aperture defined by the radiation oncologist. A custom electron block was ordered to project a light field with a uniform 1.0 cm margin around the MB aperture. In vivo film dosimetry was performed to verify adequate dose was delivered to the patient's skin and adequate sparing of the adjacent healthy tissue was achieved. Patient setup was verified by the radiation oncologist prior to treatment delivery, and Gafchromic EBT3 film was placed on the patient's surface under 0.5 cm of bolus material.

## 3 RESULTS

### 3.1 Attenuation measurements

**FIGURE 3:**
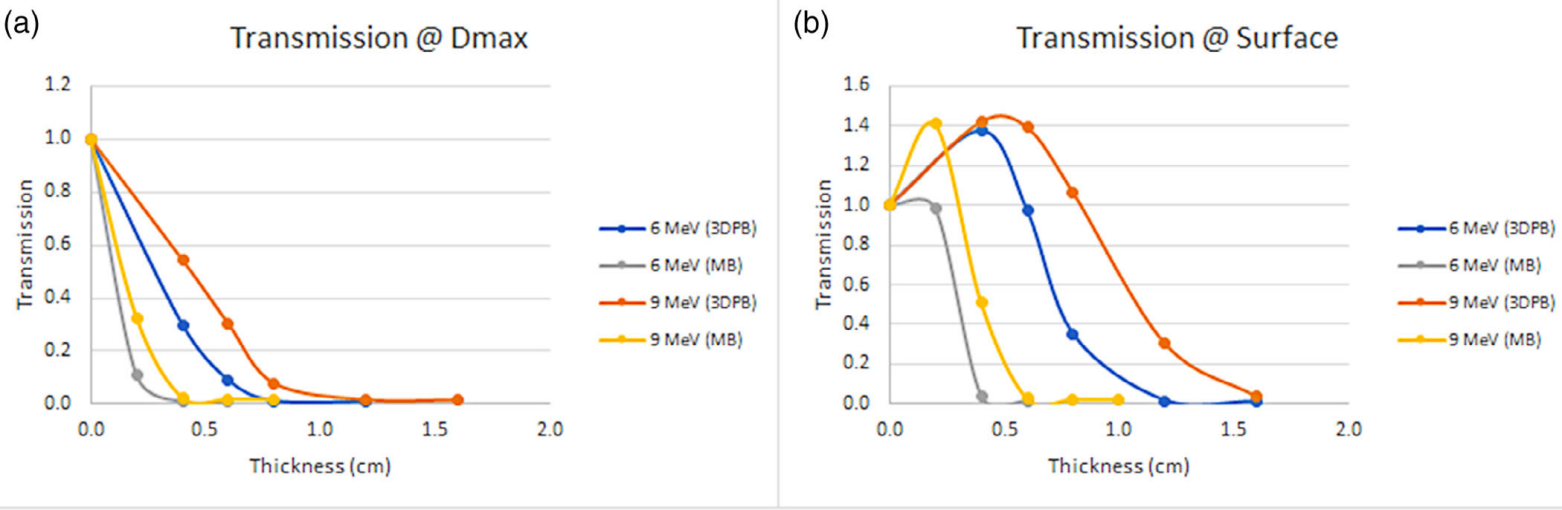
Transmission results at (a) surface and (b) dmax through varying thicknesses of 3DPB and MB. Transmission curves were used to determine the appropriate thickness of clinical surface collimators for both materials.

Attenuation results at the surface and depth of maximum dose are presented below (Figure 3). Based on these results, clinical MB thicknesses of 0.5  and 0.7 cm and clinical 3DPB thicknesses of 1.1  and 1.6 cm were chosen for 6 and 9 MeV, respectively, to minimize transmission through the surface collimators while also minimizing the weight of the skin shield that will be placed on the patient's surface. AAPM Task Group 25 recommends a transmitted dose ≤10% for external shielding, and conservative clinical thicknesses were chosen in this work to produce transmission values ≤5% at both the surface and depth of maximum dose.^12^ Note that an increase in transmission is seen at the surface due to buildup in the attenuating material, so clinical thicknesses were chosen to sufficiently attenuate the electron beams at both the surface and depth of maximum dose.

### 3.2 Relative dose plane analysis

**FIGURE 4:**
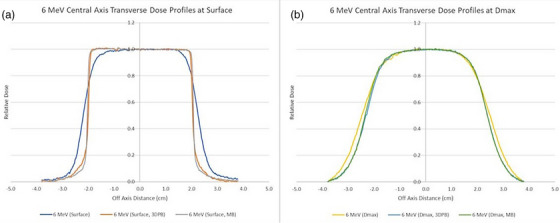
6 MeV central axis transverse dose profiles at (a) surface and (b) dmax displaying the sharper penumbras obtained near the surface for both surface collimation materials.

**FIGURE 5:**
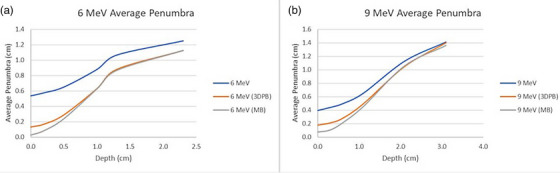
Measured average penumbra results at various depths for (a) 6 MeV and (b) 9 MeV electron beams.

The field width, flatness, and penumbra for all dose planes were calculated as the average between the transverse and radial values obtained from the relative dose profiles. The results for both 3DPB and MB surface collimators were very similar for all investigated metrics. Compared to measurements performed with no surface collimation, the flatness and penumbra results were improved when using either the 3DPB or the MB surface collimation. Transverse dose profiles at the surface and depth of maximum dose show that the improvement in dose falloff is greatest at shallower depths (Figure 4). For 6 MeV electron beams, the average penumbra values (calculated as the distance between the 80% and 20% isodose lines) ranged from 0.54 to 1.25 cm with no surface collimation, 0.13 to 1.13 cm with 3DPB surface collimation, and 0.03 to 1.13 cm with MB surface collimation (Figure 5a). For 9 MeV, the average penumbra ranged from 0.40 to 1.41 cm with no surface collimation, 0.18 to 1.40 cm with 3DPB surface collimation, and 0.08 to 1.35 cm with MB surface collimation (Figure 5b).

### 3.3 Surface dose measurements

As expected, the addition of surface collimation increases the surface dose due to increased scatter in the surface collimator compared to an equivalent beam delivery without surface collimation. For 6 MeV, relative surface doses of 1.27 and 1.14 were measured for 3DPB and MB when no bolus material was used. With the addition of 0.5 cm of bolus material, the relative surface doses decreased to 1.09 and 1.01. For 9 MeV, relative surface doses of 1.09 and 1.00 were measured for 3DPB and MB, and little change was observed in the relative surface dose values with the addition of 0.5 cm of bolus.

### 3.4 Patient treatment in vivo dosimetry

**FIGURE 6:**
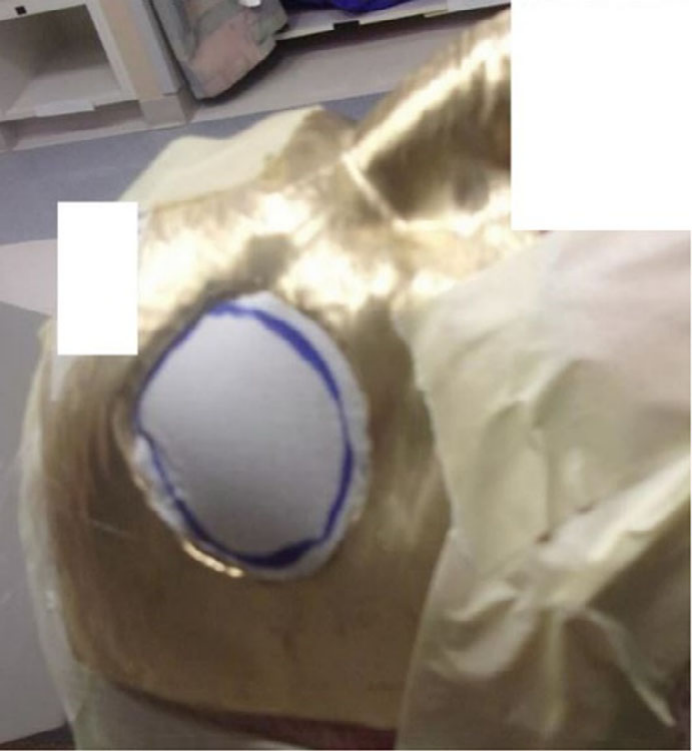
MB surface collimator placed on the patient for a right temple treatment. Bolus material was cut to fit and placed in the aperture of the MB device.

Treatment for the first patient treated at our institution with an MB device proceeded as intended with a 0.5 cm thick MB surface collimator (Figure 6). The penumbra at the field edge measured 0.2 cm, which agrees well with phantom penumbra measurements at a depth of 0.5 cm as shown in Figure 5a.

## 4 DISCUSSION

Two materials for the creation of patient‐specific skin collimators were evaluated in this work: 3DPB and MB. Both produce similar dosimetric results that create sharp dose gradients at the collimator edge, which can help minimize dose to superficial OARs near the treatment area. The only notable dosimetric difference between the two techniques is that the 3DPB generates a larger relative surface dose due to its increased thickness compared to the MB collimators. 3DPB requires a greater thickness to provide adequate shielding due to the decreased density compared to MB (3.9  vs. 8.5 g/cc). The ratio of 3DPB to MB density (8.5/3.9 = 2.2) agrees well with the ratio of clinically chosen device thicknesses (1.1/0.5 = 2.2 & 1.6/0.7 = 2.3). As discussed by Craft et al., this increase in surface dose is generally not a concern because bolus is typically used, which minimizes this effect. The treatment target is typically superficial, so the increased dose is delivered to the target. The penumbra at the surface was also slightly better with MB, potentially due to more precise fabrication resulting in a collimator with better contact with the underlying dose plane.

The primary difference between the two techniques lies in the fabrication workflows and distribution of work. In‐house 3D printing of patient‐specific devices requires significant time, effort, and expertise to maintain a 3D printing program and perform appropriate quality assurance.^14^, ^15^ At our institution, this workload is carried by the medical physics team, who are responsible for the creation of 3DPB devices and maintenance of the equipment. The vendor‐provided MB solution transfers the fabrication effort away from clinical staff and does not require the creation and upkeep of a 3D printing program. It is also worth noting that the filament used for 3D printing in this work (Bronzefill) is not a radiation oncology specific product, and the long‐term availability of the product is not guaranteed. This should be considered if clinical operations are dependent on the product.

The turnaround time for a 3DPB device is 1–2 days, depending on the print size. In comparison, the turnaround time for a MB collimator is roughly three days. Cost varies between the two techniques, with 3D printing requiring a large upfront cost to purchase a 3D printer and lesser costs in the future for materials. The vendor‐provided solution cost is greater per device with no upfront cost. Ultimately, the appropriate choice will depend on the resources available at the institution. However, either option will provide clinically acceptable results.

## 5 CONCLUSION

We have compared two different patient‐specific skin collimation techniques for electron beam radiotherapy. By assessing the costs and benefits of the two techniques and their impact on clinical workflows, institutions can make decisions that lead to increased efficiency and better patient care. As 3D printing technology continues to develop, additional high‐density filaments may be of interest for further investigation.

## AUTHOR CONTRIBUTIONS

**Steven M. Herchko**: Conceptualization of work; data acquisition; data analysis; data interpretation; draft manuscript preparation; draft manuscript revision; final approval of manuscript. **Michael S. Rutenberg**: Conceptualization of work; draft manuscript revision; final approval of manuscript. **Chris J. Beltran**: Conceptualization of work; draft manuscript revision; final approval of manuscript. **Sridhar Yaddanapudi**: Conceptualization of work; data acquisition; data analysis; data interpretation; draft manuscript preparation; draft manuscript revision; final approval of manuscript.

## CONFLICT OF INTEREST STATEMENT

The authors declare no conflicts of interest.
